# Anifrolumab for Nonsystemic Cutaneous Lupus Erythematosus: Clinical Experience, Immunologic Insights, and Review of the Literature

**DOI:** 10.3390/jcm14165683

**Published:** 2025-08-11

**Authors:** Javier Loricera, Carmen Bejerano, Andrea Estébanez, Irene García, Nasser Mohammad, Mireia Sanmartín, Marta González-Fernández, Iván Ferraz Amaro, Marcos A. González-López, Mayra V. García-Contreras, Marcos López-Hoyos, Ricardo Blanco

**Affiliations:** 1 Department of Rheumatology, Hospital Universitario Marqués de Valdecilla, 39008 Santander, Spain; cbejerano@gmail.com (C.B.); ricardo.blanco@scsalud.es (R.B.); 2 Immunopathology Group, IDIVAL, 39011 Santander, Spain; marcosantonio.gonzalez@scsalud.es (M.A.G.-L.); mayravanessa.garcia@scsalud.es (M.V.G.-C.); marcos.lopez@scsalud.es (M.L.-H.); 3 Department of Dermatology, Hospital Universitario Doctor Peset, 46017 Valencia, Spain; andreaestebanez_7@hotmail.com; 4 Department of Dermatology, Hospital del Mar, 08003 Barcelona, Spain; igarciadiez@hmar.cat (I.G.); nmohammad@hmar.cat (N.M.); 5 Department of Rheumatology, Hospital General Universitario de Valencia, 46014 Valencia, Spain; mireia.sanmartinez@gmail.com; 6 Department of Rheumatology, Hospital Universitario de Basurto, 48013 Bilbao, Spain; marta12-3-93@hotmail.com; 7 Department of Rheumatology, Complejo Hospitalario Universitario de Canarias, 38320 Tenerife, Spain; iferrazamaro@hotmail.com; 8 Department of Dermatology, Hospital Universitario Marqués de Valdecilla, 39008 Santander, Spain; 9 Department of Immunology, Hospital Universitario Marqués de Valdecilla, 39008 Santander, Spain; 10 Molecular Biology Department, University of Cantabria, 39011 Santander, Spain

**Keywords:** anifrolumab, cutaneous lupus, chronic cutaneous lupus erythematosus, subacute cutaneous lupus erythematosus, chilblain lupus, lupus tumidus, monocytes, TLRs, NK cells

## Abstract

**Objective:** Anifrolumab is approved for systemic lupus erythematosus (SLE). Its off-label use in non-systemic cutaneous lupus erythematosus (NSCLE) remains poorly characterized. We aimed to assess its effectiveness and safety in refractory NSCLE, supported by a literature review and exploratory immunologic analysis. **Methods:** This multicenter observational study included patients with NSCLE treated with anifrolumab. Skin disease was assessed using the Cutaneous Lupus Erythematosus Disease Area and Severity Index (CLASI). CLASI scores at baseline were compared to months 1, 3, and 6. A narrative literature review was also conducted. In a subset of three patients, peripheral blood immunophenotyping was performed before and after treatment to explore immunologic surrogate markers associated with clinical response. **Results:** Fifteen patients (11 women; mean age 52.1 ± 11.7 years) were included. All had received topical corticosteroids and hydroxychloroquine. Most of them had failed multiple systemic therapies. Anifrolumab (300 mg IV every 4 weeks) was used in combination (*n* = 12) or as monotherapy (*n* = 3). All patients improved. Median CLASI-A decreased from 16 to 1 (*p* < 0.001); CLASI-D decreased from 5 to 4 (*p* < 0.001). The literature review identified 6 publications reporting 14 additional cases of NSCLE with similar outcomes and minimal adverse effects. Immunologic profiling pointed to an increase in intermediate and non-classical and decreased PD-1 expression in monocytes and NK cells after 12 weeks of treatment. **Conclusions**: Anifrolumab appears effective and relatively safe in refractory NSCLE. Preliminary immunologic data suggest changes in peripheral blood monocyte subsets and NK cells. However, these findings must be confirmed in prospective, controlled clinical trials.

## 1. Introduction

Systemic lupus erythematosus (SLE) is a chronic autoimmune disease affecting multiple organs, including the skin. Cutaneous manifestations are among the most frequent clinical features of SLE [1]. However, cutaneous lupus erythematosus (CLE) also occurs in patients without systemic involvement, referred to as non-systemic CLE (NSCLE).

According to Gilliam and Sontheimer, CLE is classified into three major forms: acute CLE (ACLE), subacute CLE (SCLE), and chronic CLE (CCLE) [2]. CCLE encompasses several subtypes, with discoid lupus being the most common, and also comprises lupus panniculitis and hypertrophic discoid lupus. Variants such as lupus tumidus and chilblain lupus are less easily classified.

Historically, CLE treatment has relied on topical agents, antimalarials, corticosteroids, and conventional synthetic disease-modifying antirheumatic drugs (DMARDs). Advances in lupus pathogenesis have led to the development of biologic DMARDs, expanding therapeutic strategies.

Interferons (IFNs) are cytokines involved in immune regulation, T-cell activation, and inflammation. They are categorized into three major types—type I, type II, and type III—based on structure, function, and cellular source. Type I IFNs include 13 IFN-α subtypes and single genes for IFN-β, IFN-ε, IFN-κ, and IFN-ω. Type II IFN consists exclusively of IFN-γ. Type III IFNs belong to the interleukin-10 (IL-10) superfamily and include IFN-λ1 (IL-29), IFN-λ2 (IL-28A), IFN-λ3 (IL-28B), and IFN-λ4 [3].

IFNs are elevated in CLE and SLE, with type I IFN-related gene overexpression reported particularly in CCLE and SCLE [4]. Type I IFNs signal through a heterodimeric receptor (IFNAR) composed of two subunits, IFNAR1 and IFNAR2, expressed on all nucleated cells. Ligand binding induces dimerization and activates intracellular kinases such as Janus kinase 1 (JAK1) and tyrosine kinase 2 (TYK2) [3].

Anifrolumab is a fully human monoclonal antibody targeting IFNAR1. It is approved for the treatment of adults with moderate to severe SLE. By blocking the type I IFN receptor, anifrolumab inhibits downstream signaling, reducing interferon-stimulated gene expression and modulating inflammation. The phase 3 TULIP-1 and TULIP-2 trials demonstrated its efficacy and safety in active SLE [5]. In TULIP-2, 49% of patients achieved ≥ 50% improvement in CLASI-A scores after 52 weeks of therapy [6].

The type I IFN signature has been associated with an increased frequency of circulating intermediate (CD14CD16+) and non-classical (CD14+CD16) monocytes in SLE. Anifrolumab treatment has shown a reduction in intermediate monocytes, which may serve as an immunological marker of clinical response in CLE [7].

The therapeutic value of anifrolumab in CLE patients not meeting SLE classification criteria remains unclear. Available evidence originates mostly from systemic disease cohorts, while NSCLE data are limited to isolated case reports and small series. To address this gap, we conducted a multicenter retrospective study to evaluate the effectiveness and safety of anifrolumab in patients with NSCLE. In addition, we performed a narrative literature review and exploratory immunologic analysis to contextualize our findings within the broader clinical landscape.

## 2. Materials and Methods

### 2.1. Patients, Enrollment Criteria, and Study Protocol

We conducted an observational, open-label, multicenter study involving 15 patients diagnosed with subacute CLE (SCLE), chronic CLE (CCLE), lupus tumidus, and/or chilblain lupus, treated with anifrolumab in real-world clinical practice. Prior to anifrolumab initiation, all patients had received topical therapies and antimalarials; some had also received conventional synthetic and/or biologic immunosuppressive agents. To reduce selection bias, all individuals who had received at least one dose of anifrolumab were included, regardless of clinical outcome.

All patients were diagnosed by an experienced dermatologist, and none met the classification criteria for systemic lupus erythematosus (SLE) [8]. CLE diagnosis was confirmed by skin biopsy in all cases.

Systemic infections and malignancies were ruled out prior to treatment in accordance with national guidelines for the use of biologic therapies in rheumatic diseases [9,10,11,12]. Anifrolumab was administered intravenously at the standard dose of 300 mg every four weeks. It was prescribed due to lack of efficacy of and/or intolerance to conventional therapies. In all cases, anifrolumab was used off-label, since it is currently approved only for SLE by the EMA and FDA. Written informed consent was obtained from all patients.

This study was conducted in compliance with STROBE guidelines for observational research [13].

### 2.2. Clinical Definitions, Laboratory Data, and Outcomes

Patients with SCLE presented with annular lesions showing peripheral extension and central clearing or with papulosquamous/psoriasiform plaques [14]. CCLE included classic discoid lupus erythematosus (DLE)—characterized by well-defined erythematous papules or plaques with scaling, often leading to scarring, depigmentation, and telangiectasia [15]—as well as hypertrophic DLE and lupus panniculitis [1]. Other variants included chilblain lupus, manifesting as violaceous or erythematous lesions on acral sites [16], and lupus tumidus, presenting as smooth-surfaced, edematous plaques in sun-exposed areas [17].

Antinuclear antibodies (ANA) were detected via indirect immunofluorescence (IIF) on HEp-2 cells (1:80 dilution); titers ≥ 1:160 were considered positive. Anti-SSA/Ro and anti-SSB/La antibodies were evaluated using double immunodiffusion and/or ELISA. Anti-double-stranded DNA antibodies were assessed via IIF with *Crithidia luciliae* and by ELISA.

Disease activity and damage were measured using the Cutaneous Lupus Erythematosus Disease Area and Severity Index (CLASI). Activity (CLASI-A, range 0–70) and damage (CLASI-D, range 0–56) scores were recorded [18]. The primary outcome was the change in CLASI-A and CLASI-D scores over time.

### 2.3. Immunologic Analysis

In a subset of three patients, peripheral blood samples were collected immediately before the first anifrolumab dose and again after 12 weeks of treatment. Immunologic profiling was performed to investigate biological responses to anifrolumab, focusing on immune cell populations and interferon-related pathways.

Peripheral blood mononuclear cells (PBMCs) were isolated and analyzed by multiparametric flow cytometry to quantify B and T lymphocytes, natural killer (NK) cells, and monocyte subsets. The expression of checkpoint inhibitors—including programmed cell death protein-1 (PD-1) and T-cell immunoreceptor with Ig and ITIM domains (TIGIT)—was assessed, given their association with disease activity in SLE. Additionally, expression of Toll-like receptors (TLRs) 1–9 and intracellular cytokine production following TLR stimulation were evaluated.

Levels of intracellular cytokine-producing cells together with surface expression were determined by DX-Flex cytometry (Beckman Coulter, Brea, CA, USA) to quantify the numbers of the different T CD4^+^ subsets. Percentages of Th subsets were referred to the CD4^+^ T cells. Surface staining was performed as previously described [19,20].

### 2.4. Data Collection

Data were extracted from clinical records using a standardized protocol, reviewed for accuracy, and entered into a secure electronic database. All entries were double-checked to prevent transcription errors.

### 2.5. Statistical Analysis

Demographic and clinical variables were expressed as mean ± standard deviation (SD) or as percentages for categorical data. For non-normally distributed variables, median and interquartile range (IQR) were used. Changes in CLASI scores over time were analyzed with Wilcoxon’s signed-rank test.

All statistical analyses were performed using Stata software, version 17/SE (StataCorp, College Station, TX, USA). A *p*-value < 0.05 was considered statistically significant.

### 2.6. Literature Review

To contextualize our findings and assess current evidence regarding anifrolumab in CLE, we conducted a narrative review. PubMed and Embase databases were searched up to June 2025 using the following terms: “anifrolumab”, “cutaneous lupus erythematosus”, “discoid lupus”, “SCLE”, “CCLE”, and “skin lupus”.

We included clinical trials, observational studies, case series, and reports describing anifrolumab use in CLE, with or without SLE. Titles and abstracts were screened, and full-text articles were reviewed for relevance. References from selected studies were examined for additional sources.

Studies were included if they reported clinical data on effectiveness or safety of anifrolumab in CLE, regardless of patient age, subtype, or treatment setting. Reports focusing solely on systemic manifestations without cutaneous outcomes were excluded.

This review aimed to summarize current evidence and compare published outcomes with those observed in our cohort of NSCLE patients.

### 2.7. Ethical Considerations

**Figure 1:**
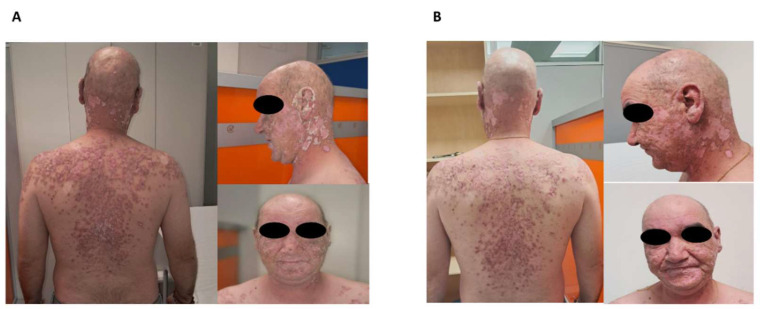
Clinical images of a patient with refractory discoid lupus erythematosus who had previously failed treatment with high-potency topical glucocorticoids and hydroxychloroquine. (**A**) Before initiation of anifrolumab therapy. (**B**) Four weeks after the first intravenous infusion of anifrolumab (300 mg), showing marked improvement in erythema and lesion infiltration.

This study was approved by the Cantabria Clinical Research Ethics Committee (internal code 2023.417; approval date: 8 March 2024) and conducted in compliance with the Declaration of Helsinki and ICH Good Clinical Practice guidelines. All data extracted from the medical records were stored and de-identified prior to the analysis. As per the Clinical Research Ethics Committee, this retrospective research did not require informed consent, except the informed consent for the taking and subsequent publication of the photographs of the patient in Figure 1.

## 3. Results

### 3.1. Baseline Features at Anifrolumab Initiation

We included 15 patients (11 women) with a mean age of 52.1 ± 11.7 years, all diagnosed with CLE and treated with anifrolumab. None met classification criteria for SLE. Patient characteristics are summarized in Table 1.

The distribution of CLE subtypes was as follows:

- CCLE (*n* = 10; 66.7%), including DLE (*n* = 9; 60%), lupus panniculitis (*n* = 1; 6.7%), and chronic hypertrophic lupus (*n* = 1; 6.7%)
- SCLE (*n* = 5; 33.3%)
- Other CLE variants (*n* = 2; 13.3%), including chilblain lupus (*n* = 1; 6.7%) and lupus tumidus (*n* = 1; 6.7%)

Three patients presented with overlapping forms of CLE. All diagnoses were confirmed by skin biopsy.

Skin lesions were most commonly located on the face (*n* = 13; 86.7%), neck (*n* = 10; 66.7%), neckline (*n* = 10; 66.7%), arms (*n* = 10; 66.7%), scalp (*n* = 7; 46.7%), hands (*n* = 8; 53.3%), trunk (*n* = 6; 40%), and lower limbs (*n* = 5; 33.3%). Alopecia was observed in 7 patients (46.7%). Arthralgia was reported in eight patients (53.3%) and asthenia in seven (46.7%).

Regarding immunological markers, eight patients (53.3%) had positive antinuclear antibodies, eight (53.3%) had anti-Ro/SSA antibodies, and two (13.3%) had anti-La/SSB antibodies. Three patients (20%) had positive anti-double-stranded DNA antibodies without fulfilling SLE criteria.

The median disease duration prior to anifrolumab initiation was 31 months [IQR 15.5–133]. All patients had previously received topical glucocorticoids (100%) and hydroxychloroquine (100%). Other prior treatments included methotrexate (*n* = 11; 73.3%), oral glucocorticoids (*n* = 10; 66.7%), chloroquine (*n* = 4; 26.7%), mycophenolate mofetil (*n* = 4; 26.7%), mepacrine (*n* = 3; 20%), azathioprine (*n* = 3; 20%), apremilast (*n* = 2; 13.3%), belimumab (*n* = 2; 13.3%), rituximab (*n* = 2; 13.3%), thalidomide (*n* = 1; 6.7%), and enpatoran in a clinical trial (*n* = 1; 6.7%).

### 3.2. Anifrolumab Effectiveness

**Figure 2:**
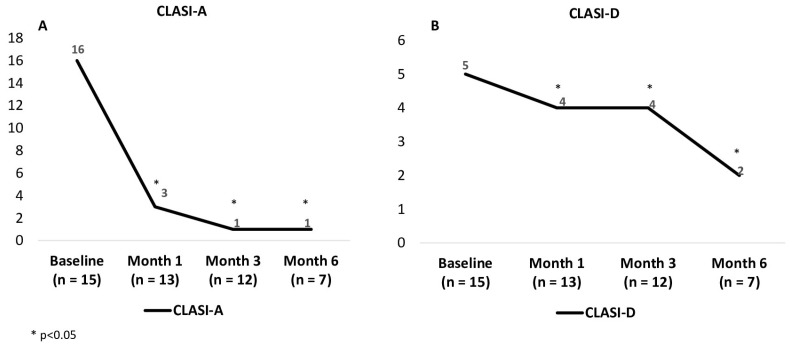
(**A**) Evolution of CLASI-A (Cutaneous Lupus Erythematosus Disease Area and Severity Index—Activity) scores in 15 patients with non-systemic cutaneous lupus treated with anifrolumab. (**B**) Evolution of CLASI-D (Damage) scores in the same cohort over the course of treatment.

Anifrolumab was administered at a dose of 300 mg intravenously every 4 weeks, either in combination with other therapies (*n* = 12) or as monotherapy (*n* = 3) (Table 1). All patients showed rapid and substantial improvement in cutaneous disease activity, as measured by CLASI-A and CLASI-D scores (Table 1; Figure 1 and Figure 2).

During a mean follow-up of 6.1 ± 4.1 months, the median CLASI-A score decreased from 16 [IQR 9.5–24.5] to 1 [IQR 0–2.5] (*p* < 0.001), and the median CLASI-D score decreased from 5 [IQR 2.5–9.5] to 4 [IQR 1.5–6.5] (*p* < 0.001). One patient discontinued anifrolumab after two doses due to the disappearance of skin lesions and worsening of asthenia with this drug.

### 3.3. Safety

Three patients (20%) experienced significant adverse events during follow-up: one developed bacterial cellulitis, and another experienced septic shock secondary to gastroenteritis. One patient reported worsening of asthenia. These patients had no known immunodeficiencies or predisposing conditions that could favor the occurrence of side effects. No patient had herpes infection during follow-up.

### 3.4. Immunologic Analysis

Data from three patients showed an increase in the numbers of intermediate and non-classical monocytes in peripheral blood after the 12th week of treatment with anifrolumab. We explored the expression of TIGIT and PD-1 in circulating immune cells, as they have been found associated with activity of SLE, especially in NK cells [21,22]. There was no significant change for TIGIT, but a decrease was detected for PD-1 in both monocytes (the three subsets) and NK cells. On the other hand, no changes in TLR expression and increased production of IL-1β, but neither IL-6 nor TNF-α after TLR stimulation, was detected. We did not observe significant findings in Th1, Th2, or Th17 cell counts.

### 3.5. Findings from the Literature Review

**Figure 3:**
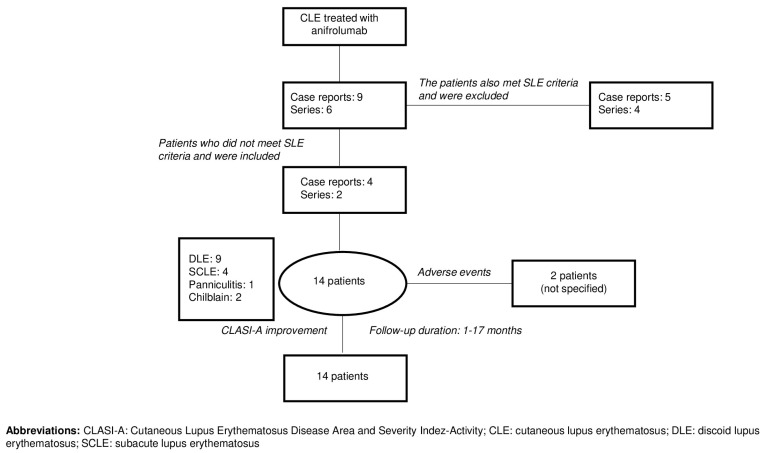
Flow chart of patient selection and evolution of the patients with CLE treated with anifrolumab from the literature review.

Our literature review identified six relevant publications describing the use of anifrolumab in patients with CLE who did not meet SLE classification criteria [23,24,25,26,27,28]. These included case reports and small case series, with a total of 14 patients. Most cases involved refractory DLE, often associated with alopecia, mucosal involvement, or hypertrophic variants. Anifrolumab was administered at the standard intravenous dose of 300 mg every 4 weeks, either as monotherapy or in combination with other immunosuppressants. Figure 3 reflects the flow chart summarizing patient selection and evolution of the patients.

Across the reviewed cases, clinical improvement was typically observed within the first 1–3 months of treatment. Several reports documented reductions in CLASI-A scores and resolution of inflammation, erythema, and pruritus. In some cases, hair regrowth and repigmentation of lesions were also noted. Adverse events were rare and generally mild.

Compared to our cohort of 15 patients, the literature cases were more heterogeneous in terms of CLE subtype and prior treatments. However, both sources consistently reported rapid and sustained improvement in cutaneous disease activity with anifrolumab. Table 2 summarizes the key differences and similarities.

These findings support the growing body of evidence that anifrolumab is effective and well tolerated in patients with CLE without systemic involvement. While our study provides the largest real-world cohort to date, the literature adds valuable clinical context, particularly in refractory or atypical presentations [23,24,25,26,27,28].

## 4. Discussion

This study provides real-world data on the effectiveness and safety of anifrolumab in 15 patients with NSCLE, including chronic, subacute, tumidus, and chilblain subtypes. To our knowledge, this is the largest clinical series to date evaluating anifrolumab in CLE without SLE. All patients showed clinical improvement, with a rapid and sustained reduction in disease activity (CLASI-A) and a modest but significant decrease in damage scores (CLASI-D), supporting its potential role in refractory disease.

Our findings are consistent with those reported in the literature. We identified six publications describing 14 additional patients with NSCLE treated with anifrolumab, most of whom had failed multiple conventional therapies including antimalarials, immunosuppressants, and biologics [23,24,25,26,27,28]. Across these reports, clinical responses were generally favorable, with improvement often observed within the first 1–3 months of treatment. Adverse events were infrequent and mild, aligning with the safety profile observed in our cohort.

In addition, exploratory immunologic analysis in three patients revealed changes consistent with the known mechanism of action of anifrolumab. Specifically, we found that intermediate and non-classical monocytes increased after treatment with no additional changes in other circulating immune cells. There is only one previous report in the literature that shows a normalization in the number of intermediate monocytes in a seven-patient series [7]. We looked for additional subsets and markers. No important findings were observed in the expression of TLRs with an isolated increased production of IL-1β after TLR stimulation. Importantly, we detected a decrease after treatment in the expression of the checkpoint inhibitor molecule PD-1 in monocytes and NK cells and no change in TIGIT. Both PD-1 and TIGIT have been described as associated with clinical activity in SLE. No data have been reported previously in CLE. In any case, our findings in monocyte subsets and NK cells point to monocytes and NK cells with a more active phenotype (PD-1 decreased expression). Our limitation is that this finding is from only three patients and no definite conclusions should be obtained. In addition, the study of the immune cells addressed here in skin biopsies should be more informative. The increase of intermediate and non-classical monocytes or the decreased expression of an inhibitor such as PD-1 could be a counterbalanced response to the tissue specific response.

SLE is a chronic autoimmune and inflammatory disorder that affects multiple organ systems due to complex immunological alterations. It most commonly affects women between puberty and menopause [29,30]. CLE is a recognized subset of lupus that primarily affects the skin and may occur either in isolation or in association with SLE.

CLE is typically classified into acute (ACLE), subacute (SCLE), and chronic (CCLE) forms, with DLE being the most common subtype of CCLE [2]. Other cutaneous variants that are more difficult to classify include lupus tumidus and chilblain lupus.

Photoprotection and smoking cessation are essential components of CLE management. Therapeutic strategies for CLE are largely adapted from those used in SLE and include topical corticosteroids, calcineurin inhibitors, antimalarials, conventional synthetic DMARDs, and biologic agents. However, these treatments are not always effective in controlling disease activity [31]. IFNs, particularly type I IFNs, have been shown to play a central role in the pathogenesis of both SLE and CLE.

Type I IFNs are a family of tightly regulated antiviral cytokines that initiate innate and adaptive immune responses [32,33]. Local production of type I IFNs is implicated in all CLE subtypes. IFN signatures are enriched in both lesional and non-lesional lupus skin, where IFN-κ, produced by keratinocytes rather than plasmacytoid dendritic cells, contributes to photosensitivity [34,35,36].

Type I IFN expression is triggered by nucleic acid sensing through intracellular pathways such as Toll-like receptor (TLR)-mediated signaling. Binding of type I IFNs to their receptors activates the JAK/STAT signaling cascade, leading to the induction of effector molecules involved in innate and adaptive immunity [3,37].

Anifrolumab is a fully human IgG1 kappa monoclonal antibody targeting the type I IFN receptor subunit 1. It is approved by the FDA for the treatment of moderate to severe SLE [30]. Anifrolumab promotes endocytosis of the type I IFN receptor, inhibits IFN-dependent STAT1 phosphorylation, and reduces type I IFN production [38].

Its efficacy has been demonstrated in the phase 2b MUSE trial and the phase 3 TULIP-1 and TULIP-2 trials, where intravenous anifrolumab 300 mg every 4 weeks showed benefit across multiple clinical endpoints [39,40,41,42]. Notably, anifrolumab led to sustained reductions in oral glucocorticoid use, fewer disease flares, and improvements in CLASI scores.

In the TULIP trials, over 45% of patients achieved a ≥50% reduction in CLASI-A scores after 52 weeks of treatment [40,41,42,43]. A post hoc analysis suggested that clinical improvement in cutaneous symptoms may begin as early as 8 weeks [40].

However, anifrolumab is not approved for patients with CLE who do not meet SLE criteria. For this reason, available data on anifrolumab in CLE are predominantly derived from patients meeting SLE criteria [26,44,45,46,47,48,49,50,51,52]. Nevertheless, available data on its use in isolated CLE are limited to case reports and one small series [23,24,25,26,27,28]. These include patients with refractory DLE, alopecia, mucosal involvement, and hypertrophic or tumidus variants, many of whom showed rapid and sustained improvement following anifrolumab initiation.

For example, Hernández-Salas et al. have just published a series of seven patients with CLE (five with CCLE and two with SCLE). This series consists of three additional patients meeting SLE criteria [27]. The authors observed a rapid improvement in symptoms and cutaneous lesions in all patients within 2–3 weeks after first infusion. The most common adverse events objectified were respiratory tract infections, myalgia/arthralgia, and urinary tract infection which forced the patient to discontinue anifrolumab [27]. Han et al. reported a 21-year-old woman with recalcitrant DLE and alopecia who improved significantly after one month of anifrolumab therapy [23]. Aljohani described a 43-year-old woman with severe scalp DLE and alopecia who experienced marked clinical and psychological improvement, with CLASI-A decreasing from 18 to 3 and glucocorticoids successfully tapered [24]. Hojjatie et al. reported a case of hypertrophic CLE/lichen planus overlap that responded dramatically to anifrolumab combined with acitretin [25].

In our series, we observed improvement in both CLASI-A and CLASI-D scores as early as the first dose, with progressive improvement over time. Only three patients experienced adverse events during treatment, and these were not clearly attributable to anifrolumab.

Table S1 shows a comparison between our series and published cases of anifrolumab in non-systemic CLE.

Our findings suggest that anifrolumab is associated with rapid and sustained improvement in cutaneous lesions in patients with NSCLE. The main limitations of our study include its retrospective design, which may introduce bias due to missing data, and the relatively small sample size. Nonetheless, key information on effectiveness and safety was consistently documented, supporting the reliability of our findings. To our knowledge, this is the largest study to date evaluating outcomes of anifrolumab in patients with CLE without systemic involvement.

Despite these encouraging results, our study has limitations. The retrospective design and small sample size limit the strength of the conclusions. The absence of a control group precludes definitive attribution of clinical improvement to anifrolumab. Moreover, the immunologic analysis was exploratory and limited to three patients, requiring validation in larger cohorts. Nonetheless, the consistency between clinical outcomes, literature data, and immunologic findings provides a compelling rationale for further investigation.

In conclusion, anifrolumab appears to be an effective and well-tolerated option for patients with refractory NSCLE. Our findings, supported by a growing body of published cases and preliminary immunologic evidence, suggest that targeting the type I interferon pathway may offer meaningful benefit in this population. Prospective controlled studies are needed to confirm these observations and to better define the role of anifrolumab in the therapeutic landscape of cutaneous lupus.

## Figures and Tables

**Table 1:** Main features of the 15 patients with non-systemic cutaneous lupus erythematosus (NSCLE) treated with anifrolumab.

Pt	Age/Sex	CLE Type	Prior Systemic Therapies	Concomitant Treatment	CLASI-A Baseline	CLASI-A Final (mo)	% Δ CLASI-A	CLASI-D Baseline	CLASI-D Final (mo)	% Δ CLASI-D	AEs
1	61/M	DLE	HCQ 400 mg/day, enpatoran according to clinical trial protocol	None	9	0 [6]	100% ↓	14	14 [6]	0%	None
2	44/M	DLE	HCQ 400 mg/day	HCQ 400 mg/day	33	7 [6]	78.8% ↓	25	23 [6]	8% ↓	None
3	65/F	DLE	Oral GC 10 mg/day, HCQ 200 mg/day, MTX 10 mg weekly	Oral GC 5 mg/day	22	10 [4]	54.5% ↓	2	2 [4]	0%	None
4	56/F	DLE	HCQ 400 mg/day, MTX 15 mg weekly, AZA 100 mg/day, BEL 200 mg weekly, RTX 1 g × 2	HCQ 400 mg/day, AZA 100 mg/day	20	1 [14]	95% ↓	8	2 [14]	75% ↓	None
5	29/F	DLE	HCQ 400 mg/day, CQ 250 mg/day, mepacrine 100 mg/day, MTX 20 mg weekly, thalidomide 100 mg/day, apremilast 50 mg/day, BEL 200 mg weekly, RTX 1 g × 2	HCQ 400 mg/day, MTX 20 mg weekly	16	1 [14]	93.7% ↓	10	6 [14]	40% ↓	None
6	46/F	SCLE	HCQ 400 mg/day	HCQ 400 mg/day	10	1 [3]	90% ↓	4	4 [3]	0%	None
7	49/F	DLE	Oral GC 30 mg/day, HCQ 400 mg/day, CQ 250 mg/day, MTX 20 mg/day, AZA 150 mg/day, MMF 2 g/day	HCQ 400 mg/day, MMF 2 g/day	17	1 [3]	94.1% ↓	9	7 [3]	22.2% ↓	None
8	57/F	SCLE	Oral GC 30 mg/day, HCQ 400 mg/day, MTX 15 mg weekly	HCQ 400 mg/day, MTX 15 mg weekly	15	0 [3]	100% ↓	5	4 [3]	20% ↓	None
9	65/F	DLE	Oral GC 30 mg/day, HCQ 400 mg/day, CQ 250 mg/day, MTX 15 mg weekly	HCQ 400 mg/day	12	1 [6]	91.7% ↓	1	1 [6]	0%	None
10	70/F	SCLE	Oral GC 60 mg/day, HCQ 600 mg/day, MTX 10 mg weekly	Oral GC 5 mg/day, HCQ 400 mg/day	31	4 [5]	87.1% ↓	8	4 [5]	50% ↓	None
11	52/F	SCLE + chilblain	Oral GC 20 mg/day, HCQ 400 mg/day, MTX 15 mg weekly, AZA 150 mg/day, MMF 2 g/day	Oral GC 5 mg/day	8	0 [11]	100% ↓	0	0 [11]	–	None
12	39/F	DLE + panniculitis	Oral GC 30 mg/day, HCQ 400 mg/day, CQ 155 mg/day, MTX 15 mg weekly, MMF 1 g/day	None	3	0 [9]	100% ↓	3	0 [9]	100% ↓	Bacterial cellulitis
13	35/M	DLE	Oral GC 30 mg/day, HCQ 400 mg/day	Oral GC 30 mg/day, HCQ 400 mg/day	27	0 [4]	100% ↓	20	20 [4]	0%	None
14	57/M	SCLE + hypertrophic DLE	Oral GC 30 mg/day, HCQ 400 mg/day, MTX 17.5 ng weekly, apremilast 60 mg/day	Acitretin 25 mg/day	27	No data	–	3	No data	–	Septic shock, herpetic keratitis
15	56/F	Lupus tumidus	Oral GC 30 mg/day, HCQ 400 mg/day, mepacrine 100 mg/day, MTX 15 mg weekly, MMF 2 g/day	None	6	0 [1]	100% ↓	0	0 [1]	–	Worsening of asthenia

**Abbreviations:** AZA: azathioprine; BEL: belimumab; CQ: chloroquine; DLE: discoid lupus erythematosus; F: female; GC: glucocorticoids; HCQ: hydroxychloroquine; M: male; MMF: mycophenolate mofetil; mo: months; MTX: methotrexate; RTX: rituximab; SCLE: subacute CLE; AEs: adverse events; ↓: reduction; Δ: change. All patients had previously received topical GCs.

**Table 2:** Summary of published cases of anifrolumab use in non-systemic cutaneous lupus erythematosus (CLE), including CLE subtype, prior treatments, time to response, and outcomes. The final row summarizes the present series of 15 patients.

Study (Ref.)	CLE Subtype(s)	Prior Treatments	Time to Response	CLASI-A Improvement	Adverse Events	Follow-up Duration
Han et al. [23]	DLE with alopecia	Topical steroids, tacrolimus ointment, HCQ, AZA, prednisone	1 month	Yes (significant)	None	7 months
Aljohani [24]	DLE with alopecia	Topical and intralesional steroids, HCQ, MTX, AZA, belimumab, steroids	After 1st dose	CLASI-A ↓ from 18 to 3	None	6 months
Hojjatie et al. [25]	Hypertrophic CLE/LP overlap	HCQ, MTX, MMF, acitretin, belimumab, rituximab, abatacept	2 months	Yes (dramatic)	None	Not reported
Trentin et al. [26]	DLE	Antimalarials, topical steroids, docetaxel, doxorubicin hydrochloride, cyclophosphamide, conventional immunosuppressants, Janus kinase inhibitors	1 month	Yes	None	12 months
Hernández-Salas et al. [27]	DLE, SCLE	Steroids, HCQ, MTX, mepacrine, acitretin, thalidomide, dimethyl fumarate, laser, AZA,	1 month	Yes	2 patients (28.6%)	1–17 months
Garbarino et al. [28]	SCLE, chilblain lupus, panniculitis	Steroids, antimalarials, MTX, AZA, MMF, belimumab	1 month	Yes	No data	No data
Present Series	SCLE, CCLE, lupus tumidus, chilblain lupus	HCQ, MTX, MMF, AZA, steroids, others	As early as 1st dose	CLASI-A ↓ from 16 to 1	3 patients (20%)	Mean 6.1 ± 4.1 months

**Abbreviations:** AZA: azathioprine; CCLE: chronic cutaneous lupus erythematosus; CLASI-A: Cutaneous Lupus Erythematosus Disease Area and Severity Index—Activity; CLASI-D: Cutaneous Lupus Erythematosus Disease Area and Severity Index—Damage; CLE: cutaneous lupus erythematosus; DLE: discoid lupus erythematosus; HCQ: hydroxychloroquine; LP: lichen planus; MMF: mycophenolate mofetil; MTX: methotrexate; SCLE: subacute cutaneous lupus erythematosus; SLE: systemic lupus erythematosus; ↓: reduction.

## Data Availability

The authors confirm that all data underlying the findings are fully available without restriction. All relevant data are included in the paper.

## Supplementary Materials

The following supporting information can be downloaded at: https://www.mdpi.com/article/10.3390/jcm14165683/s1, Table S1: Comparison between the present cohort and published cases of anifrolumab use in non-systemic cutaneous lupus erythematosus (CLE).

## Abbreviatures

ACLE: acute cutaneous lupus erythematosus; ANA: antinuclear antibodies; AZA: azathioprine; BEL: belimumab; CCLE: chronic cutaneous lupus erythematosus; CLASI: cutaneous lupus erythematosus disease area and severity index; CLE: cutaneous lupus erythematosus; CQ: chloroquine; DLE: discoid lupus erythematosus; DMARDs: disease-modifying antirheumatic drugs; GC: glucocorticoids; HCQ: hydroxychloroquine; IFN: interferon; IIF: indirect immunofluorescence; IL: interleukin; JAK: Janus kinase; MMF: mycophenolate mofetil; MTX: methotrexate; NSCLE: non-systemic subacute and chronic cutaneous lupus erythematosus; PBMCs: peripheral blood mononuclear cells; RTX: rituximab; SCLE: subacute cutaneous lupus erythematosus; SLE: systemic lupus erythematosus; TYK: tyrosine kinase.
