# Supplementary material for: Anifrolumab for Nonsystemic Cutaneous Lupus Erythematosus: Clinical Experience, Immunologic Insights, and Review of the Literature
Source: J Clin Med. 2025 Aug 11;14(16):5683. doi: 10.3390/jcm14165683 (PMC12386681; doi:10.3390/jcm14165683)
Supplement: Supplementary file 1 [file jcm-14-05683-s001.zip › jcm-3787906-supplementary.pdf]

Table S1. Comparison between the present cohort and published cases of anifrolumab use in non-systemic cutaneous lupus erythematosus (CLE).

Feature	Our Series (n= 15)	Literature Review (n= 11; refs 21–25)
Study design	Retrospective multicenter cohort	Case reports and one small series
CLE subtypes	SCLE, CCLE, lupus tumidus, chilblain lupus	Mostly DLE, some hypertrophic, mucosal, alopecia
SLE criteria met	None	None
Median disease duration	31 months [IQR 15.5–133]	Variable (not always reported)
Prior treatments	Topical steroids, antimalarials, DMARDs	Similar; often multiple failed therapies
Anifrolumab dose	300 mg IV every 4 weeks	Same
Time to response	As early as first dose	Typically, 1–2 months
CLASI-A improvement	Median decreased from 16 to 1	In all cases
Adverse events	3 patients (20%), not clearly related to treatment	Rare; mostly absent or mild
Follow-up duration	Mean 6.1±4.1 months	Variable (1-17 months in most reports)

Abbreviations: CCLE: chronic cutaneous lupus erythematosus; CLE: cutaneous lupus erythematosus; CLASI-A: Cutaneous Lupus Erythematosus Disease Area and Severity Index – Activity; DMARDs: disease-modifying antirheumatic drugs; DLE: discoid lupus erythematosus; IV: intravenous; SCLE: subacute cutaneous lupus erythematosus; SLE: systemic lupus erythematosus.
